# Inborn Errors of Immunity Associated With Type 2 Inflammation in the USIDNET Registry

**DOI:** 10.3389/fimmu.2022.831279

**Published:** 2022-02-22

**Authors:** Kelsey L. Smith, Darlene Dai, Bhavi P. Modi, Rahnuma Sara, Elizabeth Garabedian, Rebecca A. Marsh, Jennifer Puck, Elizabeth Secord, Kathleen E. Sullivan, Stuart E. Turvey, Catherine M. Biggs, the USIDNET Consortium

**Affiliations:** ^1^ Department of Pediatrics, University of British Columbia, Vancouver, BC, Canada; ^2^ British Columbia (BC) Children’s Hospital, Vancouver, BC, Canada; ^3^ National Human Genome Research Institute, Bethesda, MD, United States; ^4^ National Institutes of Health, Bethesda, MD, United States; ^5^ Cincinnati Children’s Hospital, University of Cincinnati, Cincinnati, OH, United States; ^6^ Division of Allergy/Immunology and Blood and Marrow Transplantation, Department of Pediatrics, University of California, San Francisco, San Francisco, CA, United States; ^7^ Wayne State University, Detroit, MI, United States; ^8^ Children’s Hospital of Philadelphia, University of Pennsylvania Perelman School of Medicine, Philadelphia, PA, United States; ^9^ St Paul’s Hospital, Vancouver, BC, Canada

**Keywords:** atopy, primary immunodeficiency, IgE, eosinophilia, inborn error of immunity

## Abstract

**Background:**

Monogenic conditions that disrupt proper development and/or function of the immune system are termed inborn errors of immunity (IEIs), also known as primary immunodeficiencies. Patients with IEIs often suffer from other manifestations in addition to infection, and allergic inflammation is an increasingly recognized feature of these conditions.

**Methods:**

We performed a retrospective analysis of IEIs presenting with allergic inflammation as reported in the USIDNET registry. Our inclusion criteria comprised of patients with a reported monogenic cause for IEI where reported lab eosinophil and/or IgE values were available for the patient prior to them receiving potentially curative therapy. Patients were excluded if we were unable to determine the defective gene underlying their IEI. Patients were classified as having eosinophilia or elevated IgE when their record included at least 1 eosinophil count or IgE value that was greater than the age stratified upper limit of normal. We compared the proportion of patients with eosinophilia or elevated IgE with the proportion of samples in a reference population that fall above the upper limit of normal (2.5%).

**Results:**

The query submitted to the USIDNET registry identified 1409 patients meeting inclusion criteria with a monogenic cause for their IEI diagnosis, of which 975 had eosinophil counts and 645 had IgE levels obtained prior to transplantation or gene therapy that were available for analysis. Overall, 18.8% (183/975) of the patients evaluated from the USIDNET registry had eosinophilia and 20.9% (135/645) had an elevated IgE. IEIs caused by defects in 32 genes were found to be significantly associated with eosinophilia and/or an elevated IgE level, spanning 7 of the 10 IEI categories according to the International Union of Immunological Societies classification.

**Conclusion:**

Type 2 inflammation manifesting as eosinophilia or elevated IgE is found in a broad range of IEIs in the USIDNET registry. Our findings suggest that allergic immune dysregulation may be more widespread in IEIs than previously reported.

## Introduction

Monogenic conditions that disrupt proper development and/or function of the immune system are termed inborn errors of immunity (IEIs), also known as primary immunodeficiencies (1, 2). IEIs are heritable conditions with more than 400 genetically unique forms (3). Early diagnosis of IEIs is critical, as timely intervention can improve outcomes and prevent mortality and morbidity (2). Clinical heterogeneity amongst IEIs contributes to delayed diagnosis, which can span several years, complicating treatment and increasing the risk of serious infection (4–6). Depending on the underlying molecular defect, those with IEIs may suffer from infection, autoimmunity, inflammation and/or cancer (7–9). Allergic inflammation is an increasingly acknowledged manifestation of immune dysregulation in IEI (10). This subcategory of IEI conditions associated with type 2 inflammation can be referred to as Primary Atopic Disorders (PADs) (11).

PADs involve an underlying interplay between sufficient effector cells for an allergic response, with inadequate protection from infection (11). Allergic inflammation, however, may be the dominating or sole clinical manifestation when a patient initially seeks medical care, highlighting the importance of allergy providers in evaluating for PADs (10, 12). Identifying patients suffering from PADs is crucial so that they may have access to life-changing treatments such as hematopoietic stem cell transplantation, immunoprophylaxis and/or targeted biologics/small molecules tailored to their specific molecular defect (1, 7, 13, 14). Eosinophilia and elevated serum IgE levels are two laboratory indicators of type 2 allergic inflammation, and are classically associated with PADs such as Wiskott-Aldrich syndrome (WAS), dedicator of cytokinesis 8 (DOCK8) deficiency, and immune dysregulation, polyendocrinopathy, enteropathy, X-linked (IPEX) syndrome (15–19).

As the number of known monogenic immune disorders increase, it is important to identify which IEIs are associated with allergic inflammation and could be considered a PAD (20). To investigate this, we queried laboratory results from patients enrolled in the United States Immunodeficiency Network (USIDNET) registry with a diagnosed monogenic IEI and available eosinophil and IgE laboratory data. The USIDNET registry is a source of clinical and laboratory data on patients with IEIs from across North America and has led to important discoveries on the manifestations of IEIs (21). Here we identify a number of IEIs associated with type 2 inflammation, revealing that the spectrum of PADs may be broader than previously recognized.

## Methods

### USIDNET Registry Dataset

We performed a retrospective analysis of IEIs presenting with allergic inflammation as reported in the USIDNET registry (date of data release was 5/14/2020). The USIDNET is an Immune Deficiency Foundation program that is funded by the National Institute of Allergy and Infectious Diseases (NIAID) and the National Institutes of Health (NIH). Physicians and healthcare providers from different sites across North America provide clinical data on their own patients with IEIs, which is compiled to form a large registry accessible for research. Informed consent is provided for all patients in the database. We submitted a query to the USIDNET requesting laboratory data, specifically eosinophil and IgE levels, for patients with a diagnosed IEI within the registry. Of the information provided by the USIDNET, the data fields surveyed included demographics, the patient’s IEI diagnosis and affected gene, gene variant molecular information, hematopoietic stem cell (HSC) or solid organ transplant history, gene therapy history, complete blood counts (CBCs), specifically eosinophils, immunoglobulins, specifically IgE, allergic conditions and allergic reactions.

### Identification of Eligible Case Data

**Figure 1:**
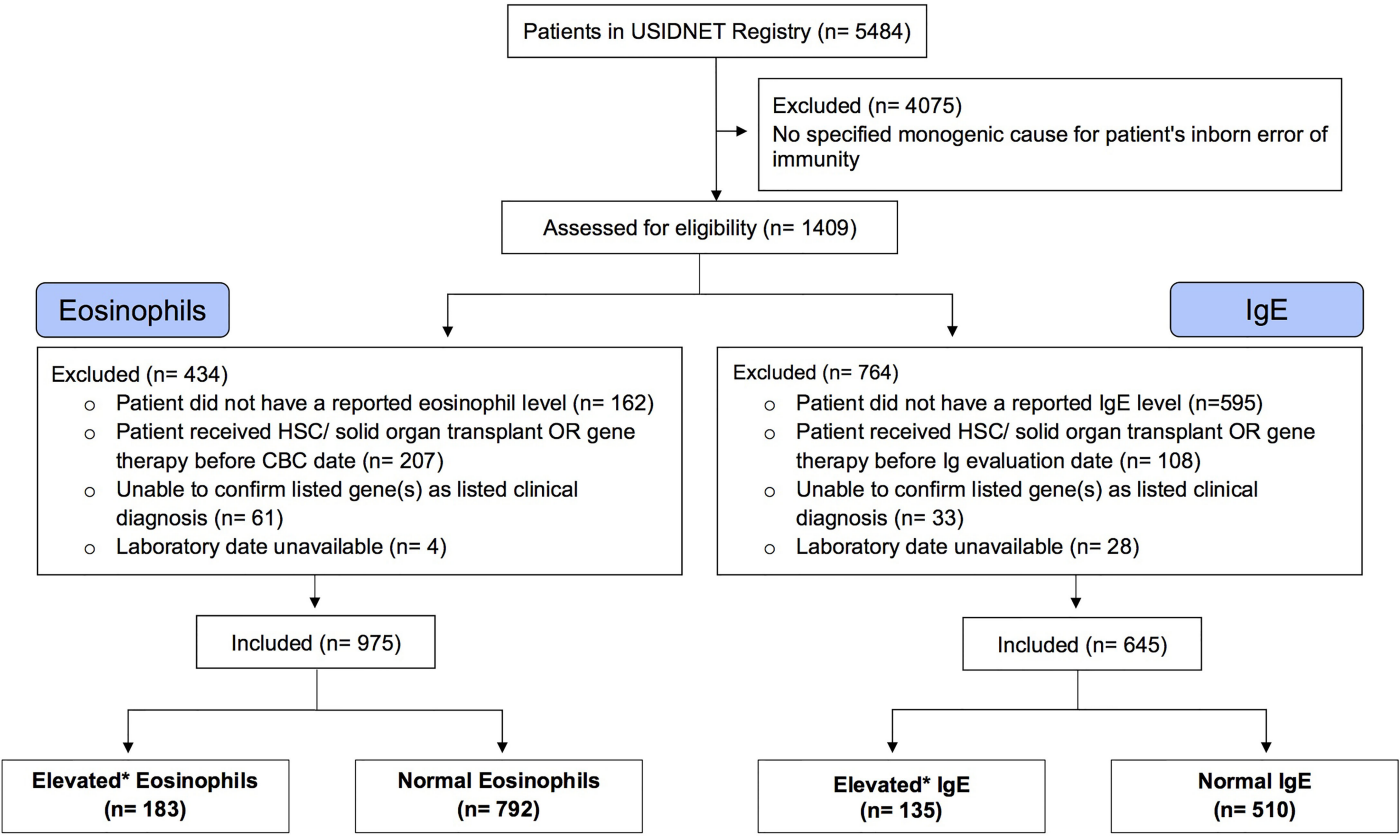
Selection of Eligible Patient Cohort. A consort diagram describing review of the USIDNET Registry data and selection of eligible patient data for analysis. USIDNET indicates United States Immunodeficiency Network; HSC, Hematopoietic stem cell; Ig, Immunoglobulin; CBC, complete blood count. *Elevated based on age-based reference interval.

The USIDNET data were reviewed and processed to create an analytical dataset for this study (**Figure 1**). Our inclusion criteria comprised of patients with a reported monogenic cause for IEI where reported lab eosinophil and/or IgE values were available for the patient prior to them receiving potentially curative therapy (either gene therapy or hematopoietic stem cell/organ transplant). Patients were excluded if we were unable to determine the defective gene underlying their IEI or they did not have a reported laboratory date. Patients were classified as having eosinophilia or elevated IgE based on the respective age stratified upper limit (22, 23). Patient age was calculated by the difference between the date that their eosinophil or IgE level was measured and June 15^th^ of their birth year, as birth month and day are not available for confidentially of the registry patients. If the difference calculated was smaller than 0, we considered the patient’s age as 0. This method of imputing the patient age minimizes error to ≤6 months.

### Identification of Reference Values

We identified two references from the literature to compare IgE and eosinophil laboratory values of our USIDNET study population. Tahmasebi and colleagues evaluated eosinophil values in 566 healthy children and adolescents under 21 years of age. This study reported the upper limit of normal for children under 1 year of age as 900 cells/μL, and the upper limit of normal for children or adolescents over the age of 1 as 500 cells/μL (22). We extrapolated the upper limit of normal for patients over the age of 21 to be 500 cells/μL. Martins and colleagues evaluated IgE in 1376 healthy children between 6 months and 17 years of age as well as 128 adults between 19 and 69 years of age (23). The upper limit of normal for IgE was stratified by age groups; 6 to 12 months, 34 IU/ml; 1 to 2 years, 97 IU/ml; 3 years, 199 IU/ml; 4 to 6 years, 307 IU/ml; 7 to 8 years, 403 IU/ml; 9 to 12 years, 696 IU/ml; 13 to 15 years, 629 IU/ml; 16 to 17 years; 537 IU/ml; 18 years and older, 214 IU/ml (23). We extrapolated the upper limit for infants less than 6 months to be 34 IU/ml.

### Statistical Analysis

Two proportion z-test was applied to compare the proportion of patients with a specific IEI who had eosinophilia, elevated IgE or both versus the proportion of samples in a reference population that fall above the upper limit of normal (2.5%). Only genes with lab records for at least 3 patients were included in the analysis. Adjusted p-values were calculated using Benjamini-Hochberg method and genes with adjusted p-value < 0.05 were selected (24). To confirm the robustness of our imputed age estimate, sensitivity analysis was performed by repeating our primary analysis with the estimated age either 6 months younger or 6 months older than June 15 of the birth year (**Supplemental Table 1**). All statistical analyses were done with R software (version 4.0.4).

## Results

**Figure 2:**
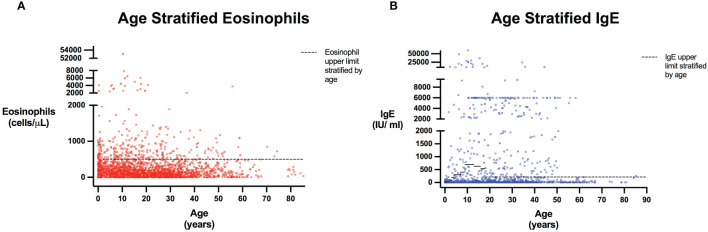
Eosinophils and IgE stratified by age. Scatter plots show all patients included in the analysis with every reported Eosinophil **(A)** or IgE **(B)** value. Patient age was calculated as the time interval between their laboratory date and June 15^th^ of their birth year, as birth month and date were not provided for patient confidentiality and anonymity. Dashed line represents Eosinophil and IgE upper limits stratified by age based on reference populations (22, 23). Eosinophil upper limits correspond to 900 cells/μL in those <1 years, and 500 cells/μL for those 1 years of age and older (with values for individuals aged greater than 21 extrapolated to be 500 cells/μL). IgE level upper limits are: 6 to 12 months, 34 IU/ml; 1 to 2 years, 97 IU/ml; 3 years, 199 IU/ml; 4 to 6 years, 307 IU/ml; 7 to 8 years, 403 IU/ml; 9 to 12 years, 696 IU/ml; 13 to 15 years, 629 IU/ml; 16 to 17 years; 537 IU/ml; 18 years and older, 214 IU/ml (23). For individuals less than 6 months of age, the IgE level upper limit was extrapolated as 34 IU/ml.

The query submitted to the USIDNET registry identified 1409 patients meeting inclusion criteria with a monogenic cause for their IEI diagnosis, of which 975 had eosinophil counts and 645 had IgE levels obtained prior to transplantation or gene therapy that were available for analysis (**Figure 1**). **Table 1** outlines the demographics of the cohort studied. We evaluated eosinophilia and elevated IgE independently and therefore a patient may be represented in one or both evaluations. Each eosinophil and IgE record was compared to the age stratified upper limit (**Figure 2**). Overall, 18.8% (183/975) of the patients evaluated from the USIDNET registry had eosinophilia and 20.9% (135/645) had elevated IgE.

**Table 1:** Demographic characteristics of selected “Eosinophils” and “IgE” cohorts.

Variable	Eosinophil	IgE
**Total Patients**	975	645
**Sex**	**n(%)**	**n(%)**
Male	665 (68.2%)	421 (65.3%)
Female	310 (31.8%)	224 (34.7%)
**Ethnicity**	**n(%)**	**n(%)**
White/Caucasian	685 (70.3%)	464 (71.9%)
Black/African American	75 (7.7%)	39 (6%)
Asian or Pacific Islander	26 (2.7%)	19 (2.9%)
American Indian/Alaska Native	2 (0.2%)	2 (0.3%)
Other or More Than One Race	52 (5.3%)	34 (5.3%)
Unknown or Not Reported	135 (13.8%)	87 (13.5%)
**Living**	**n(%)**	**n(%)**
Yes	883 (90.6%)	581 (90.1%)
No	84 (8.6%)	61 (9.4%)
Unknown	8 (0.8%)	3 (0.5%)
Age * (years)	**n(%), reference upper limit (cells/μL)**	**n(%), reference upper limit (IU/mL)**
<1	93 (9.5%), 900	59 (9.1%), 34
1-3	61 (6.3%), 500	37 (5.7%), 97
3-4	25 (2.6%), 500	14 (2.2%), 199
4-7	73 (7.5%), 500	44 (6.8%), 307
7-9	48 (4.9%), 500	30 (4.7%), 403
9-13	114 (11.7%), 500	64 (9.9%), 696
13-16	84 (8.6%), 500	48 (7.4%), 629
16-18	52 (5.3%), 500	40 (6.2%), 537
>=18	425 (43.6%), 500	309 (47.9%), 214

*Patient age was calculated by the difference between the date of the patient’s last Eosinophil or IgE record and June 15^th^ of their birth year, as exact birth month and date are not available due to confidentially and anonymity of the patients in the registry.

### IEIs Associated With Eosinophilia

**Figure 3:**
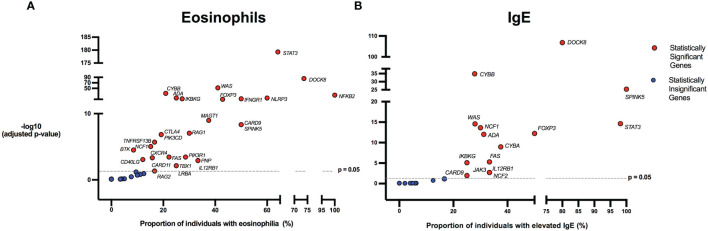
Inborn errors of immunity associated with eosinophilia and elevated IgE. Volcano plots show the proportion of eosinophilia and elevated IgE on the x-axis. The y-axis shows the -log10 of the adjusted p-value based on the two-proportion z-test, performed by comparing the proportion of eosinophilia/elevated IgE for each gene to the proportion of values in the reference population falling above the upper limit of normal (2.5%). Dotted line indicates the significance threshold of the adjusted p-value at 0.05 and genes above the line are statistically significant. HGNC gene symbols used. **(A)** IEIs caused by monogenic defects in 29 genes (red) were found to be significantly associated with eosinophilia compared to the reference population age stratified upper limit. **(B)** IEIs caused by monogenic defects in 15 genes (red) were found to be significantly associated with elevated IgE compared to the age stratified upper limit.

**Figure 4:**
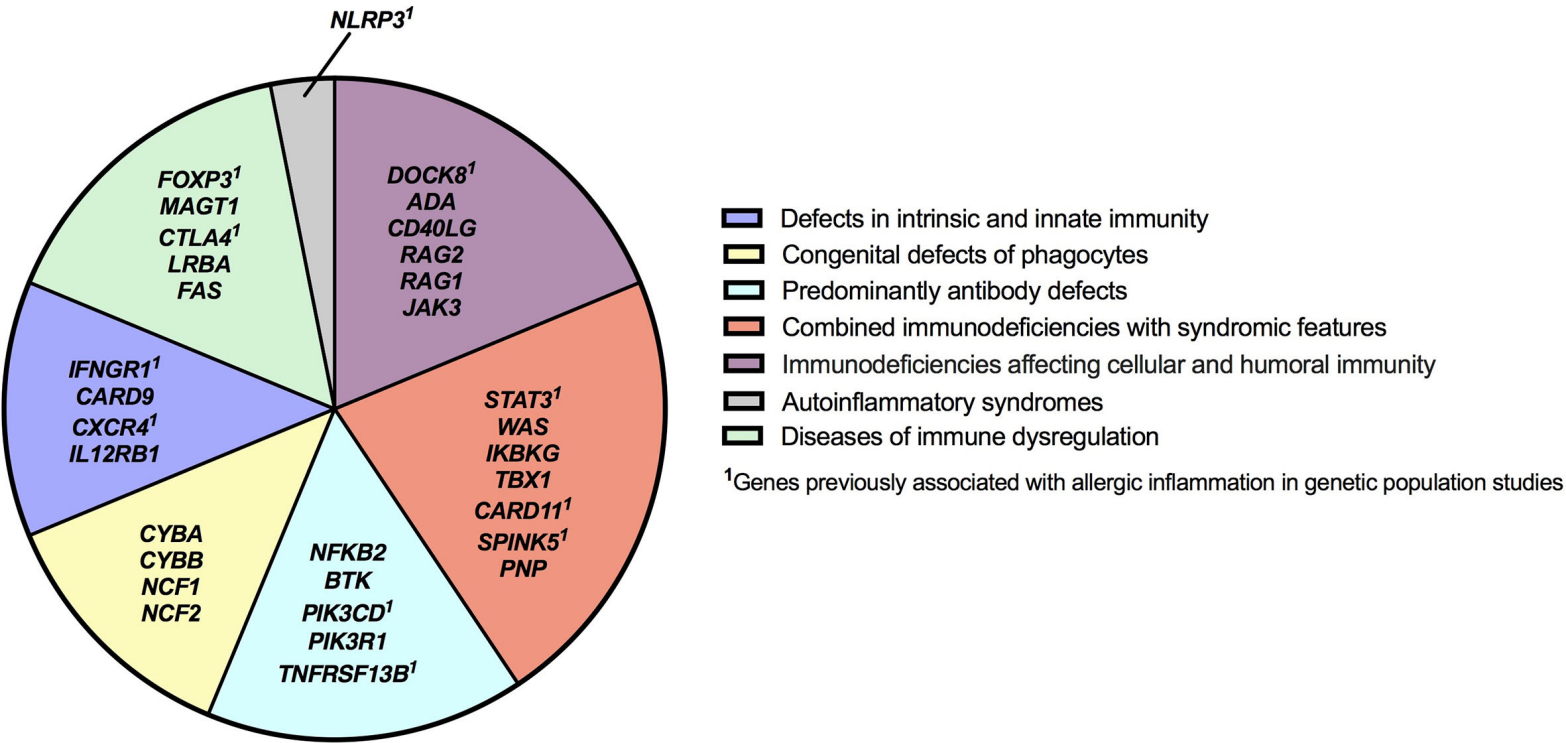
Inborn errors of immunity genes associated with type 2 inflammation by disease category. IEI genes found to be associated with type 2 inflammation in our study are categorized according to IUIS Phenotypic Classification. HGNC gene symbols are used.

**Figure 5:**
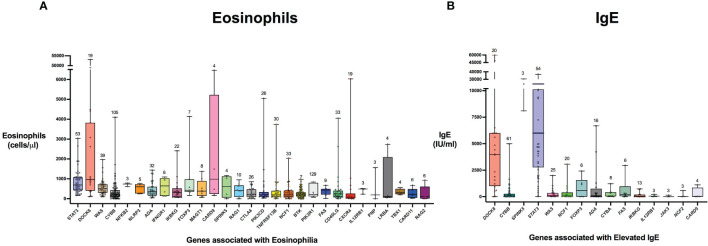
Distribution of laboratory values in inborn errors of immunity associated with type 2 inflammation compared to the reference population. The genes are ordered by adjusted p-value from lowest to highest for eosinophil count **(A)** and IgE level **(B)**. For each gene, the box plot spans the interquartile range between the upper and lower quartile, with the median laboratory value marked by the black horizontal line across the colored box plot. The whiskers on either side of the box plot show the minimum and maximum laboratory values. The value above each whisker indicates the number of patients for each IEI that were evaluated for eosinophilia or IgE. HGNC gene symbols used.

There were 975 patients meeting inclusion criteria who had eosinophil data prior to undergoing transplantation or gene therapy available for analysis. To determine which IEIs are associated with eosinophilia, we evaluated each monogenic IEI’s proportion of patients in the registry with eosinophilia compared to 2.5%, which is the proportion of values falling above the age stratified upper limit of normal in the reported reference population (22). With correction for multiple testing, IEIs caused by defects in the following 29 genes were significantly associated with eosinophilia: *STAT3, DOCK8, WAS, CYBB, NFKB2, NLRP3, ADA, IFNGR1, IKBKG, FOXP3, MAGT1, CARD9, SPINK5, RAG1, CTLA4, PIK3CD, TNFRSF13B, NCF1, PIK3R1, BTK, FAS, CD40LG, CXCR4, IL12RB1, PNP, LRBA, TBX1, CARD11*, and *RAG2* (**Figure 3**). As these results were determined by imputing birth month to June 15^th^ of the known birth year, sensitivity analysis was performed by repeating the primary analysis twice, using an estimated birth month 6 months younger or older than June 15^th^. The results were unchanged except for *TBX1*, which remained significant in one of the two analyses (**Supplemental Table 1**). These conditions spanned 7 of the 10 categories of the International Union of Immunological Societies (IUIS) classification for IEIs (**Figure 4**). Within these IEIs, the proportion of patients with eosinophilia ranged from 8.5 to 100%, with the highest proportion identified in *NFKB2* (**Figure 3** and **Supplemental Table 1**). The eosinophil value ranged from 0 to 53100 cells/μL, with the highest value identified in *DOCK8* (**Figure 5**). Moderate eosinophilia (above 1500 cells/ μL) was observed in *PNP, LRBA, TNFRSF13B, CD40LG, IKBKG, NCF1, FOXP3, WAS, STAT3, CYBB*, and *DOCK8*, with severe eosinophilia (above 5000 cells/ μL) observed in *CXCR4, PIK3CD, CARD9* and *DOCK8* (**Figure 5** and **Supplemental Table 1**). We then evaluated the registry data to look for reported allergic clinical disease in these same patients whose IEI genes were found to be significantly associated with eosinophilia. Among the 29 IEI genes associated with eosinophilia, 27 had at least one allergic clinical manifestation (eczema, asthma, allergic rhinitis, or food allergy) present in 10% or more of patients (**Supplemental Table 1**).

### IEIs Associated With Elevated IgE

In order to evaluate IEIs associated with an elevated IgE, we studied 645 of the 1409 USIDNET patients who met study inclusion criteria and had an IgE value obtained prior to undergoing transplantation or gene therapy with a reported IgE evaluation date. We evaluated each monogenic IEI’s proportion of patients in the registry with an elevated IgE compared to 2.5%, which is the proportion of values falling above the age stratified upper limit of normal in the reported reference population (23). With correction for multiple testing, IEIs caused by defects in the following 15 genes were significantly associated with elevated IgE: *DOCK8, CYBB, SPINK5, STAT3, WAS, NCF1, FOXP3, ADA, CYBA, IKBKG, FAS, IL12RB1, JAK3, NCF2*, and *CARD9* (**Figures 3**, **4**). All of these genes were significant in sensitivity analysis (**Supplemental Table 1**). Within these IEIs, the proportion of patients with an elevated IgE ranged from 23 to 100%, with the highest proportion identified in *SPINK5* (**Figure 3** and **Supplemental Table 1**). The IgE value ranged from 0 to 60000 IU/ml, with the highest proportion identified in *DOCK8* (**Figure 5**). We then evaluated the registry data to look for reported allergic clinical disease in these same patients whose IEI genes were found to be significantly associated with elevated IgE. At least one allergic clinical manifestation (eczema, asthma, allergic rhinitis, or food allergy) was found in 10% or more of patients belonging to 13 of the 15 IEI gene categories (**Supplemental Table 1**).

### IEIs Associated With Eosinophilia and Elevated IgE

Building on these insights, we then sought to determine which of these IEIs were associated with elevations in both eosinophil and IgE levels. A total of 612 patients meeting inclusion criteria had both eosinophil and IgE levels obtained prior to transplantation or gene therapy. Among them, 72 (12%) patients had both eosinophilia and elevated IgE, and eosinophilia was significantly associated with elevated IgE by fisher exact test (p < 0.001, **Supplemental Table 2**). We evaluated each IEI gene’s proportion of patients in the registry with elevations in both eosinophil and IgE levels compared to 2.5%, which is the proportion of patients in the age stratified reference populations that fall above the upper limit (22, 23). After correcting for multiple testing, IEIs caused by defects in the following 10 genes were associated with both eosinophilia and elevated IgE levels: *ADA, CARD9, CYBB, DOCK8, FOXP3, IKBKG, NCF1, SPINK5, STAT3, WAS*. Ten percent or more of patients from each category had at least one clinical allergic manifestation (eczema, asthma, allergic rhinitis or food allergy) (**Supplemental Table 1**).

## Discussion

IEIs are often difficult diagnoses to make, requiring careful consideration of the patient’s personal and family history, physical exam findings, as well as laboratory and genetic testing. Patients may suffer complications for several years before the definitive investigations are performed (6, 25). Diagnostic delay can be attributed to many reasons including barriers to genetic testing, the perception that recurrent infections or allergy are expected components of childhood, insufficient awareness around clinical presentations of IEIs, as well as the lack of professional guidance on recognizing and managing these patients in primary care (25, 26). Consequences of delayed recognition may include suboptimal management, unnecessary complications, inaccurate genetic counselling, and anguish for both the patient and their family (27). PADs may be particularly difficult to identify owing to the high prevalence of polygenic allergic disease (28–30). Clinical red flags for PADs that have been suggested in the literature include severe atopy, autoimmunity, malignancy, connective tissue disease, growth failure/short stature, and recurrent infections (31, 32). In this study we looked for laboratory evidence of type 2 inflammation in the USIDNET registry and found that approximately one in five patients with an IEI attributable to a monogenic defect had eosinophilia and/or an elevated IgE level.

Determining which IEIs are significantly associated with type 2 inflammation is critical to inform the differential diagnosis of clinicians evaluating patients with allergic disease. It also provides powerful insights into particular genes and pathways that may be implicated in driving allergic immune dysregulation. This could lead to new therapeutic targets for both IEIs and polygenic allergic conditions. Furthermore, type 2 inflammation may be more than a laboratory indicator for allergic phenotypes. Eosinophilia and elevated IgE could potentially serve as diagnostic clues of an underlying IEI, and future studies should evaluate the time course between laboratory findings of allergic inflammation, presenting clinical features, and IEI diagnosis.

Our results show that type 2 inflammation spans multiple categories of IEIs from combined immunodeficiencies to autoinflammatory disease, outlining how allergic immune dysregulation occurs in a diverse range of IEIs (**Figure 4**) (3). As expected, our study identified several well-known PADs such as *STAT3, WAS, DOCK8, SPINK5*, and *CARD11* that are classified according to the IUIS as combined immunodeficiency with or without syndromic features. Mechanisms underlying the allergic immune dysregulation seen in these conditions range from impaired epithelial barrier (*SPINK5*), to actinopathy (*WAS*, *DOCK8*), to altered antigen receptor (*CARD11*) and cytokine signaling (*STAT3*). While the highest absolute eosinophil values were observed in DOCK8 deficiency, roughly one quarter of patients with this condition had eosinophil values within the normal range, highlighting that a normal eosinophil count should not exclude this IEI from ones’ differential diagnosis.

The category of immune dysregulatory syndromes contained both anticipated findings: *FOXP3* (IPEX syndrome) and *FAS* (autoimmune lympoproliferative syndrome)–both known to be associated with eosinophilia/elevated IgE–and surprises: identifying conditions in which allergic inflammation is less prominent (*CTLA4* and *LRBA*) or to our knowledge, unreported, as a clinical feature (*MAGT1*) (33–36). The transcription factor FOXP3 is critical for regulatory T cell function and the maintenance of immune tolerance; allergists should be familiar with IPEX syndrome, as patients may initially present with dermatitis, and some are diagnosed with allergic diseases before the condition is recognized (37). Interestingly, several genes identified in our study including *CTLA4*, *STAT3*, *IFNGR1, FOXP3, DOCK8, SPINK5, PIK3CD, TNFRSF13B, CXCR4, CARD11* and *NLRP3* have been reported as candidate genes for allergic disease in genetic population studies (**Figure 4**) (38–46).

An unexpected finding was the association of type 2 inflammation with genes implicated in Chronic Granulomatous disease (CGD). CGD is typically characterized by neutrophilic granulocyte and monocyte impairment predisposing patients to recurrent and severe infection, autoimmunity, and dysregulated inflammation (47). In our study, we found associations with 4 CGD genes, *CYBA, CYBB*, *NCF1* and *NCF2*, with *CYBB* previously linked to allergic inflammation in the literature (48). While peripheral eosinophilia has been rarely reported in CGD, tissue eosinophilia is a known feature. Examples include eosinophilic infiltrates in CGD-associated inflammatory bowel disease and eosinophilic cystitis. In one reported case of peripheral eosinophilia in CGD, the patient’s eosinophil levels normalized after successfully treating a *Nocardia nova* infection, which is a known pathogen associated with CGD and eosinophilia (48). This highlights the possibility that some IEIs may be associated with peripheral eosinophilia or elevated IgE because of specific infections that affected patients are predisposed to. Another possibility in CGD is that deficient NADPH oxidase activity causes skewing towards type 2 inflammation; this possibility is supported a gp91^phox-/-^ mouse model of allergic asthma, which showed increased peripheral and tissue eosinophils and production of Th2-specific cytokines compared to wild-type mice following allergen challenge (49).

Predominately antibody deficiencies are another IUIS phenotypic category of interest where we identified 5 IEI genes associated with type 2 inflammation (*NFKB2, BTK, PIK3CD, PIK3R1, TNFRSF13B).* X-linked agammaglobulinemia (XLA) caused by pathogenic *BTK* variants typically presents clinically around 4 to 6 months of age when the IgG antibodies transferred through the placenta begin to diminish (50). Patients commonly present with oto-sino-pulmonary infections secondary to encapsulated bacteria (51). Laboratory findings in XLA patients are predominately severe hypogammaglobulinemia and absent peripheral B lymphocytes, though atypical presentations can occur (50, 51). Although counterintuitive and not consistently reported in literature, XLA patients can experience clinical and laboratory features of allergic inflammation such as allergic rhinitis, elevated IgE and/or eosinophilia (52). In particular, those with hypomorphic *BTK* variants that facilitate the production of some immunoglobulins may have an allergy phenotype with no history of severe infection (52). Kaneko and colleagues hypothesize that elevated IgE in XLA patients may be a critical marker for identifying leaky XLA phenotypes (53).

Four genes categorized as defects in intrinsic and innate immunity were identified as being significantly associated with eosinophilia: *IFNGR1*, *CARD9, CXCR4*, and *IL12RB1.* Interferon-γ (IFN-γ) receptor 1 deficiency caused by loss-of-function variants in *IFNGR1* predisposes to mycobacterial infections (54). Interestingly, *IFNGR1* variants were reported in one study as the most prevalent genetic risk factor for atopic dermatitis complicated by eczema herpeticum (ADEH+) (42). In addition, Aoki et al. identified a novel variant of the *IFNGR1* gene that was present in 6 of 89 allergic patients (bronchial asthma or allergic rhinitis) and none of the non-allergic patients. Patients with this variant were also found to have elevated serum IgE levels when compared to non-allergic counterparts (55). This association between *IFNGR1* variants and allergic inflammation may be explained by impaired IFN-γ axis signaling causing enhanced Th2 effector responses. In contrast to deficiencies in innate immunity, we also identified an association between eosinophilia and NLRP3-mediated autoinflammatory syndromes. The NLRP3 inflammasome has been implicated in human eosinophilic disorders, as well as eosinophil recruitment and Th2 cytokine production in a mouse model of asthma (56, 57).

We acknowledge that our study has several limitations. Our study is limited to patients reported in the USIDNET, and therefore it is likely that the small sample size may have influenced our statistical analysis. The numbers of patients varied by condition, for example, while *NFKB2* was associated with the highest proportion of patients with eosinophilia (100%), there were only 3 patients with this condition included in our analysis. The list of genes identified in our study is also not exhaustive, as it reflects only those conditions reported in the registry. Recently described IEIs associated with allergic inflammation such as T-bet deficiency caused by *TBX21* variants, or the actinopathy ARPC1B deficiency, are therefore not captured in this study (58, 59). Our analysis also does not distinguish loss-of-function from gain-of-function IEIs caused by defects in the same gene. Our age estimation was limited by the lack of data on birth month, however, sensitivity analysis confirmed the robustness of our imputed birth month estimate, as all of the same genes remained significant apart from *TBX1*, which was still significant in one of the two sensitivity analyses performed. In addition, due to the nature of this study being registry-based, there is no information regarding the method of testing and diagnosis of IEI. Furthermore, as with many retrospective, registry-based studies, a limitation that should be recognized is the reliance on data entered by different healthcare providers across numerous institutions which may be subject to inaccuracies. Nevertheless, the USIDNET registry has provided the opportunity to develop great insight that otherwise could not be acquired for IEIs that may present infrequently at any one center. There is a need for more studies similar to this that investigate the spectrum of clinical manifestations in IEIs and PADs in order to develop more sensitive diagnostic tools.

## Concluding Remarks

In conclusion, laboratory evidence of type 2 inflammation, whether eosinophilia or elevated IgE, amongst patients with IEIs in the USIDNET registry appears to be more common and widespread than previously reported. Given our findings, clinicians in the community including allergists, dermatologists, and primary care providers should consider an IEI when investigating eosinophilia or elevated IgE.

## Data Availability Statement

The datasets presented in this study can be found in online repositories. The names of the repository/repositories and accession number(s) can be found below: All data used within our study is freely available to researchers by placing a data query through the USIDNET Primary Immunodeficiency Diseases registry. Further information can be found at: https://usidnet.org/registry-data/.

## Author Contributions

EG, RM, JP, ES, and KES contributed to registry data collection and KLS, DD, BM, RS, and ST contributed to data analysis. KLS, DD, and CB wrote the manuscript. KLS, DD, BM, RS, EG, RM, JP, ES, KES, ST, and CB critically reviewed the manuscript. All authors contributed to the article and approved the submitted version.

## Funding

This work was supported by grants from the Canadian Institutes of Health Research (PJQ-173584 to ST and CB), Genome British Columbia (SIP007) (ST), and a Clinical & Translational Research Seed Grant from the BC Children’s Hospital Research Institute (to CB and BM). ST holds a Tier 1 Canada Research Chair in Pediatric Precision Health and the Aubrey J. Tingle Professor of Pediatric Immunology. CB is supported by a Michael Smith Health Research BC Health Professional-Investigator Award and by a Providence Healthcare Research Institute Early Career Clinician Investigator award. KLS is supported by the Canadian Society of Allergy and Clinical Immunology Summer Studentship.

## Conflict of Interest

JP receives royalties from UpToDate and her spouse is employed by and owns stock in Invitae, a gene sequencing company.

The remaining authors declare that the research was conducted in the absence of any commercial or financial relationships that could be construed as a potential conflict of interest.

## Publisher’s Note

All claims expressed in this article are solely those of the authors and do not necessarily represent those of their affiliated organizations, or those of the publisher, the editors and the reviewers. Any product that may be evaluated in this article, or claim that may be made by its manufacturer, is not guaranteed or endorsed by the publisher.

## References

[1] Notarangelo LD. Primary Immunodeficiencies. J Allergy Clin Immunol (2010) 125(2 Suppl 2):S182–94. doi: 10.1016/j.jaci.2009.07.053 20042228

[2] Turvey SE Bonilla FA Junker AK. Primary Immunodeficiency Diseases: A Practical Guide for Clinicians. Postgrad Med J (2009) 85(1010):660–6. doi: 10.1136/pgmj.2009.080630 20075404

[3] Tangye SG Al-Herz W Bousfiha A Chatila T Cunningham-Rundles C Etzioni A. Human Inborn Errors of Immunity: 2019 Update on the Classification From the International Union of Immunological Societies Expert Committee. J Clin Immunol (2020) 40(1):24–64. doi: 10.1007/s10875-019-00737-x 31953710 PMC7082301

[4] Resnick ES Moshier EL Godbold JH Cunningham-Rundles C. Morbidity and Mortality in Common Variable Immune Deficiency Over 4 Decades. Blood (2012) 119(7):1650–7. doi: 10.1182/blood-2011-09-377945 PMC3286343 22180439

[5] Bomken S van der Werff Ten Bosch J Attarbaschi A Bacon CM Borkhardt A Boztug K. Current Understanding and Future Research Priorities in Malignancy Associated With Inborn Errors of Immunity and DNA Repair Disorders: The Perspective of an Interdisciplinary Working Group. Front Immunol (2018) 9:2912. doi: 10.3389/fimmu.2018.02912 30619276 PMC6299915

[6] Branch A Modi B Bahrani B Hildebrand KJ Cameron SB Junker AK. Diverse Clinical Features and Diagnostic Delay in Monogenic Inborn Errors of Immunity: A Call for Access to Genetic Testing. Pediatr Allergy Immunol (2021) 32(8):1796–803. doi: 10.1111/pai.13571 34097760

[7] McCusker C Upton J Warrington R. Primary Immunodeficiency. Allergy Asthma Clin Immunol (2018) 14(Suppl 2):61. doi: 10.1186/s13223-018-0290-5 30275850 PMC6157160

[8] Kebudi R Kiykim A Sahin MK. Primary Immunodeficiency and Cancer in Children; A Review of the Literature. Curr Pediatr Rev (2019) 15(4):245–50. doi: 10.2174/1573396315666190917154058 PMC7040504 31530267

[9] Allenspach E Torgerson TR. Autoimmunity and Primary Immunodeficiency Disorders. J Clin Immunol (2016) 36(Suppl 1):57–67. doi: 10.1007/s10875-016-0294-1 27210535

[10] Biggs CM Lu HY Turvey SE. Monogenic Immune Disorders and Severe Atopic Disease. Nat Genet (2017) 49(8):1162–3. doi: 10.1038/ng.3925 28747751

[11] Milner JD. Primary Atopic Disorders. Annu Rev Immunol (2020) 38:785–808. doi: 10.1146/annurev-immunol-042718-041553 32126183

[12] Sokol K Milner JD. The Overlap Between Allergy and Immunodeficiency. Curr Opin Pediatr (2018) 30(6):848–54. doi: 10.1097/MOP.0000000000000697 30407976

[13] Pai SY. Treatment of Primary Immunodeficiency With Allogeneic Transplant and Gene Therapy. Hematol Am Soc Hematol Educ Program (2019) 2019(1):457–65. doi: 10.1182/hematology.2019000052 PMC6913427 31808905

[14] Alvarez B Arcos J Fernández-Guerrero ML. Pulmonary Infectious Diseases in Patients With Primary Immunodeficiency and Those Treated With Biologic Immunomodulating Agents. Curr Opin Pulm Med (2011) 17(3):172–9. doi: 10.1097/MCP.0b013e3283455c0b 21415752

[15] Park JH Lee KH Jeon B Ochs HD Lee JS Gee HY. Immune Dysregulation, Polyendocrinopathy, Enteropathy, X-Linked (IPEX) Syndrome: A Systematic Review. Autoimmun Rev (2020) 19(6):102526. doi: 10.1016/j.autrev.2020.102526 32234571

[16] Williams KW Milner JD Freeman AF. Eosinophilia Associated With Disorders of Immune Deficiency or Immune Dysregulation. Immunol Allergy Clin North Am (2015) 35(3):523–44. doi: 10.1016/j.iac.2015.05.004 PMC4688016 26209898

[17] Biggs CM Keles S Chatila TA. DOCK8 Deficiency: Insights Into Pathophysiology, Clinical Features and Management. Clin Immunol (2017) 181:75–82. doi: 10.1016/j.clim.2017.06.003 28625885 PMC5555255

[18] Orange JS Stone KD Turvey SE Krzewski K. The Wiskott-Aldrich Syndrome. Cell Mol Life Sci (2004) 61(18):2361–85. doi: 10.1007/s00018-004-4086-z PMC11138727 15378206

[19] Candotti F. Clinical Manifestations and Pathophysiological Mechanisms of the Wiskott-Aldrich Syndrome. J Clin Immunol (2018) 38(1):13–27. doi: 10.1007/s10875-017-0453-z 29086100

[20] Lyons JJ Milner JD. Primary Atopic Disorders. J Exp Med (2018) 215(4):1009–22. doi: 10.1084/jem.20172306 PMC5881472 29549114

[21] Michniacki TF Connelly JA Sturza J Merz LE Marsh R Dale D. Neutropenia Is an Underrecognized Finding in Pediatric Primary Immunodeficiency Diseases: An Analysis of the United States Immunodeficiency Network Registry. J Pediatr Hematol Oncol (2020) 42(7):e601-e605. doi: 10.1097/MPH.0000000000001744 32049770

[22] Tahmasebi H Higgins V Bohn M Hall A Adeli K. CALIPER Hematology Reference Standards (I): Improving Laboratory Test Interpretation in Children (Beckman Coulter DxH 900–Core Laboratory Hematology System). Am J Clin Pathol (2020) 154(3):330–41. doi: 10.1093/ajcp/aqaa059 PMC7403759 32561916

[23] Martins TB Bandhauer ME Bunker AM Roberts WL Hill HR. New Childhood and Adult Reference Intervals for Total IgE. J Allergy Clin Immunol (2014) 133(2):589–91. doi: 10.1016/j.jaci.2013.08.037 24139495

[24] Benjamini Y Hochberg Y. Controlling the False Discovery Rate: A Practical and Powerful Approach to Multiple Testing. J R Stat Soc (1995) 57(1):289–300. doi: 10.1111/j.2517-6161.1995.tb02031.x

[25] Condino-Neto A Espinosa-Rosales FJ. Changing the Lives of People With Primary Immunodeficiencies (PI) With Early Testing and Diagnosis. Front Immunol (2018) 9:1439. doi: 10.3389/fimmu.2018.01439 29997619 PMC6031256

[26] Modell V Orange JS Quinn J Modell F. Global Report on Primary Immunodeficiencies: 2018 Update From the Jeffrey Modell Centers Network on Disease Classification, Regional Trends, Treatment Modalities, and Physician Reported Outcomes. Immunol Res (2018) 66(3):367–80. doi: 10.1007/s12026-018-8996-5 29744770

[27] Chapel H Prevot J Gaspar HB Español T Bonilla FA Solis L. Primary Immune Deficiencies - Principles of Care. Front Immunol (2014) 5:627. doi: 10.3389/fimmu.2014.00627 25566243 PMC4266088

[28] Dell SD Foty RG Gilbert NL Jerret M To T Walter SD. Asthma and Allergic Disease Prevalence in a Diverse Sample of Toronto School Children: Results From the Toronto Child Health Evaluation Questionnaire (T-CHEQ) Study. Can Respir J (2010) 17(1):e1-6. doi: 10.1155/2010/913123 20186360 PMC2866206

[29] Schernhammer ES Vutuc C Waldhör T Haidinger G. Time Trends of the Prevalence of Asthma and Allergic Disease in Austrian Children. Pediatr Allergy Immunol (2008) 19(2):125–31. doi: 10.1111/j.1399-3038.2007.00597.x 18086231

[30] Nicolaou N Siddique N Custovic A. Allergic Disease in Urban and Rural Populations: Increasing Prevalence With Increasing Urbanization. Allergy (2005) 60(11):1357–60. doi: 10.1111/j.1398-9995.2005.00961.x 16197466

[31] Vaseghi-Shanjani M Smith KL Sara RJ Modi BP Branch A Sharma M. Inborn Errors of Immunity Manifesting as Atopic Disorders. J Allergy Clin Immunol (2021) 148(5):1130–9. doi: 10.1016/j.jaci.2021.08.008 34428518

[32] Ponsford MJ Klocperk A Pulvirenti F Dalm VASH Milota T Cinetto F. Hyper-IgE in the Allergy Clinic–When is it Primary Immunodeficiency? Allergy (2018) 73(11):2122–36. doi: 10.1111/all.13578 30043993

[33] Ravell JC Chauvin SD He T Lenardo M. An Update on XMEN Disease. J Clin Immunol (2020) 40(5):671–81. doi: 10.1007/s10875-020-00790-x PMC7369250 32451662

[34] Egg D Rump IC Mitsuiki N Rojas-Restrepo J Maccari ME Schwab C. Therapeutic Options for CTLA-4 Insufficiency. J Allergy Clin Immunol (2021) 149(2):736–46. doi: 10.1016/j.jaci.2021.04.039 34111452

[35] Navabi B Upton JE. Primary Immunodeficiencies Associated With Eosinophilia. Allergy Asthma Clin Immunol (2016) 12:27. doi: 10.1186/s13223-016-0130-4 27222657 PMC4878059

[36] Butt D Chan TD Bourne K Hermes JR Nguyen A Statham A. FAS Inactivation Releases Unconventional Germinal Center B Cells That Escape Antigen Control and Drive IgE and Autoantibody Production. Immunity (2015) 42(5):890–902. doi: 10.1016/j.immuni.2015.04.010 25979420

[37] Jamee M Zaki-Dizaji M Lo B Abolhassani H Aghamahdi F Mosavian M. Clinical, Immunological, and Genetic Features in Patients With Immune Dysregulation, Polyendocrinopathy, Enteropathy, X-Linked (IPEX) and IPEX-Like Syndrome. J Allergy Clin Immunol Pract (2020) 8(8):2747–2760.e7. doi: 10.1016/j.jaip.2020.04.070 32428713

[38] Queiroz GA da Silva RR Pires AO Costa RDS Alcântara-Neves NM da Silva TM. New Variants in NLRP3 Inflammasome Genes Increase Risk for Asthma and. Cytokine X (2020) 2(3):100032. doi: 10.1016/j.cytox.2020.100032 33015616 PMC7522708

[39] Hitomi Y Ebisawa M Tomikawa M Imai T Komata T Hirota T. Associations of Functional NLRP3 Polymorphisms With Susceptibility to Food-Induced Anaphylaxis and Aspirin-Induced Asthma. J Allergy Clin Immunol (2009) 124(4):779–85.e6. doi: 10.1016/j.jaci.2009.07.044 19767079

[40] Bijanzadeh M Mahesh PA Ramachandra NB. An Understanding of the Genetic Basis of Asthma. Indian J Med Res (2011) 134:149–61. PMC3181014 21911966

[41] Howard TD Postma DS Hawkins GA Koppelman GH Zheng SL Wysong AK. Fine Mapping of an IgE-Controlling Gene on Chromosome 2q: Analysis of CTLA4 and CD28. J Allergy Clin Immunol (2002) 110(5):743–51. doi: 10.1067/mai.2002.128723 12417883

[42] Gao L Bin L Rafaels NM Huang L Potee J Ruczinski I. Targeted Deep Sequencing Identifies Rare Loss-of-Function Variants in IFNGR1 for Risk of Atopic Dermatitis Complicated by Eczema Herpeticum. J Allergy Clin Immunol (2015) 136(6):1591–600. doi: 10.1016/j.jaci.2015.06.047 PMC4679503 26343451

[43] Schaarschmidt H Ellinghaus D Rodríguez E Kretschmer A Baurecht H Lipinski S. A Genome-Wide Association Study Reveals 2 New Susceptibility Loci for Atopic Dermatitis. J Allergy Clin Immunol (2015) 136(3):802–6. doi: 10.1016/j.jaci.2015.01.047 25865352

[44] Marques CR Costa RS Costa GNO da Silva TM Teixeira TO de Andrade EMM. Genetic and Epigenetic Studies of FOXP3 in Asthma and Allergy. Asthma Res Pract (2015) 1:10. doi: 10.1186/s40733-015-0012-4 27965764 PMC5142332

[45] Astle WJ Elding H Jiang T Allen D Ruklisa D Mann AL. The Allelic Landscape of Human Blood Cell Trait Variation and Links to Common Complex Disease. Cell (2016) 167(5):1415–1429.e19. doi: 10.1016/j.cell.2016.10.042 27863252 PMC5300907

[46] Chen MH Raffield LM Mousas A Sakaue S Huffman JE Moscati A. Trans-Ethnic and Ancestry-Specific Blood-Cell Genetics in 746,667 Individuals From 5 Global Populations. Cell (2020) 182(5):1198–1213.e14. doi: 10.1016/j.cell.2020.06.045 32888493 PMC7480402

[47] Arnold DE Heimall JR. A Review of Chronic Granulomatous Disease. Adv Ther (2017) 34(12):2543–57. doi: 10.1007/s12325-017-0636-2 PMC5709447 29168144

[48] Nguyen A Patel K Puck J Dorsey M. Longstanding Eosinophilia in a Case of Late Diagnosis Chronic Granulomatous Disease. J Clin Immunol (2017) 37(2):101–3. doi: 10.1007/s10875-016-0361-7 27966181

[49] Banerjee ER Henderson WR. Defining the Molecular Role of Gp91phox in the Immune Manifestation of Acute Allergic Asthma Using a Preclinical Murine Model. Clin Mol Allergy (2012) 10(1):2. doi: 10.1186/1476-7961-10-2 22216879 PMC3266200

[50] Shillitoe BMJ Gennery AR. An Update on X-Linked Agammaglobulinaemia: Clinical Manifestations and Management. Curr Opin Allergy Clin Immunol (2019) 19(6):571–7. doi: 10.1097/ACI.0000000000000584 31464718

[51] Suri D Rawat A Singh S. X-Linked Agammaglobulinemia. Indian J Pediatr (2016) 83(4):331–7. doi: 10.1007/s12098-015-2024-8 26909497

[52] Cinicola B Uva A Leonardi L Moratto D Giliani S Carsetti R. Case Report: A Case of X-Linked Agammaglobulinemia With High Serum IgE Levels and Allergic Rhinitis. Front Immunol (2020) 11:582376. doi: 10.3389/fimmu.2020.582376 33224144 PMC7674281

[53] Kaneko H Kawamoto N Asano T Mabuchi Y Horikoshi H Teramoto T. Leaky Phenotype of X-Linked Agammaglobulinaemia in a Japanese Family. Clin Exp Immunol (2005) 140(3):520–3. doi: 10.1111/j.1365-2249.2005.02784.x PMC1809400 15932514

[54] van de Vosse E van Dissel JT. IFN-γr1 Defects: Mutation Update and Description of the IFNGR1 Variation Database. Hum Mutat (2017) 38(10):1286–96. doi: 10.1002/humu.23302 28744922

[55] Aoki M Matsui E Kaneko H Inoue R Fukao T Watanabe M. A Novel Single-Nucleotide Substitution, Leu 467 Pro, in the Interferon-Gamma Receptor 1 Gene Associated With Allergic Diseases. Int J Mol Med (2003) 12(2):185–91. doi: 10.3892/ijmm.12.2.185 12851715

[56] Lin H Li Z Lin D Zheng C Zhang W. Role of NLRP3 Inflammasome in Eosinophilic and Non-Eosinophilic Chronic Rhinosinusitis With Nasal Polyps. Inflammation (2016) 39(6):2045–52. doi: 10.1007/s10753-016-0442-z 27614764

[57] Ma M Li G Qi M Jiang W Zhou R. Inhibition of the Inflammasome Activity of NLRP3 Attenuates HDM-Induced Allergic Asthma. Front Immunol (2021) 12:718779. doi: 10.3389/fimmu.2021.718779 34413860 PMC8369415

[58] Kuijpers TW Tool ATJ van der Bijl I de Boer M van Houdt M de Cuyper IM. Combined Immunodeficiency With Severe Inflammation and Allergy Caused by ARPC1B Deficiency. J Allergy Clin Immunol (2017) 140(1):273–277.e10. doi: 10.1016/j.jaci.2016.09.061 27965109

[59] Yang R Weisshaar M Mele F Benhsaien I Dorgham K Han J. High Th2 Cytokine Levels and Upper Airway Inflammation in Human Inherited T-Bet Deficiency. J Exp Med (2021) 218(8):e20202726. doi: 10.1084/jem.20202726 34160550 PMC8225679

