# Supplementary material for: Inborn Errors of Immunity Associated With Type 2 Inflammation in the USIDNET Registry
Source: Front Immunol. 2022 Feb 22;13:831279. doi: 10.3389/fimmu.2022.831279 (PMC8902297; doi:10.3389/fimmu.2022.831279)
Supplement: Supplementary file 2 [file Table_2.docx]

|  | Not Elevated IgE | Elevated IgE |
| --- | --- | --- |
| Not Eosinophilia | 411 (67%) | 59 (10%) |
| Eosinophilia | 70 (11%) | 72 (12%) |

Supplementary Table 2. Contingency table of eosinophilia and elevated IgE for patients meeting inclusion criteria with both eosinophil counts and IgE levels obtained.
